# The impact of hip fracture on health-related quality of life and activities of daily living: the SPARE-HIP prospective cohort study

**DOI:** 10.1007/s11657-019-0607-0

**Published:** 2019-05-29

**Authors:** D. Prieto-Alhambra, D. Moral-Cuesta, A. Palmer, I. Aguado-Maestro, M. F. Bravo Bardaji, F. Brañas, G. Adrados Bueno, J. R. Caeiro-Rey, I. Andrés Cano, M. Barres-Carsi, L. Gracia Delgado, M. Salomó-Domènech, I. Etxebarria-Foronda, B. Llado Ferrer, S. Mills, L. Ezquerra Herrando, D. Mifsut, L. D. R. Evangelista, X. Nogués, I. Perez-Coto, J. Martínez-Iñiguez Blasco, C. Martín-Hernández, H. Kessel, J. Teixidor Serra, J. Rodriguez Solis, O. Torregrosa Suau, E. Vaquero-Cervino, C. Pablos Hernández, L. Rodríguez Mañas, A. Herrera, A. Díez-Perez

**Affiliations:** 1 GREMPAL (Grup de Recerca en Epidemiologia de les Malalties Prevalents de l’Aparell Locomotor) Research Group, CIBERFES, IDIAP Jordi Gol (Universitat Autònoma de Barcelona) and Instituto de Salud Carlos III, Av Gran Via de les Corts Catalanes, 587, Atic, 08007 Barcelona, Spain; 2 Musculoskeletal Pharmaco and Device Epidemiology - Centre for Statistics in Medicine, Nuffield Department of Orthopaedics, Rheumatology, and Musculoskeletal Sciences, University of Oxford, Botnar Research Centre, Windmill Road, Oxford, OX3 7LD UK; 3 Musculoskeletal Research Unit, IMIM-Parc Salut Mar, CIBERFES, Universitat Autònoma de Barcelona, Doctor Aiguader 88, 08003 Barcelona, Spain; 4 Geriatric Unit, Hospital Universitario La Paz, Paseo de la Castellana, 261, 28046 Madrid, Spain; 5 Traumatology and Orthopaedics Unit, Nuffield Orthopedic Centre, Windmill Rd, Headington, Oxford, OX3 7HE UK; 6 Hospital Universitario Rio Hortega, Calle Dulzaina, 2, 47012 Valladolid, Spain; 7 Hospital Regional Universitario de Malaga, Av. de Carlos Haya, s/n, 29010 Málaga, Spain; 8 Geriatric Unit, Hospital Universitario Infanta Leonor, Gran Vía del Este, 80, 28031 Madrid, Spain; 9 Internal Medicine Unit, Hospital Infanta Cristina, Av. de Elvas, s/n, 06080 Badajoz, Spain; 10 Traumatology and Orthopaedics Unit, Complejo Hospitalario Universitario de Santiago de Compostela, Rúa da Choupana, s/n, 15706 Santiago de Compostela, A Coruña Spain; 11 Hospital Puerta del Mar, Av. Ana de Viya, 21, 11009 Cádiz, Spain; 12 Hospital Universitari i Politècnic La Fe, Av de Fernando Abril Martorell, 106, 46026 València, Spain; 13 Hospital Universitario Reina Sofía de Cordoba, Av Menendez Pidal, 14004 Córdoba, Spain; 14 Corporación sanitaria Universitaria Parc Tauli, Parc Taulí, 1, 08208 Sabadell, Barcelona Spain; 15 Department of Orthopaedic, Alto Deba Hospital, Arrasate-Mondragon, Gipuzkoa Spain; 16 Hospital Son Llàtzer, Carretera de Manacor, PQ 4 (Son Ferriol), 07198 Palma de Mallorca, Spain; 17 Traumatology and Orthopaedics Unit, Hospital Universitario La Paz, Paseo de la Castellana, 261, 28046 Madrid, Spain; 18 F.E.A of the Traumatology and Orthopaedics Unit, Hospital Clínico Universitario Lozano Blesa, Av. San Juan Bosco, 15, 50009 Zaragoza, Spain; 19 Hospital Clínico de Valencia, Av de Blasco Ibáñez, 17, 46010 Valencia, Spain; 20 Geriatric Unit, Hospital Universitario Severo Ochoa, Av. De Orellana s/n, 28911 Leganés, Madrid Spain; 21 Internal Medicine Department IMIM (Hospital del Mar Medical Research), CIBER FES ISCIII, Universitat Autónoma de Barcelona, Barcelona, Spain; 22 Hospital Universitario San Agustín, Camino de Heros, 6, 33401 Avilés, Asturias Spain; 23 Hospital San Pedro, Calle Piqueras, 98, 26006 Logroño, La Rioja Spain; 24 IIS Aragón (Instituto de Investigación Sanitaria de Aragón), Hospital Universitario Miguel Servet, Paseo Isabel la Católica 1-3, 50009 Zaragoza, Spain; 25 Geriatric Care Unit, Complejo Hospitalario Torrecárdenas, Calle Hermandad de Donantes de Sangre, 04009 Almería, Spain; 26 Hospital Universitari Vall de Hebron, Passeig de la Vall d’Hebron, 119-129, 08035 Barcelona, Spain; 27 Geriatric Unit, Hospital Universitario de Guadalajara, Calle Donante de Sangre, s/n, 19002 Guadalajara, Spain; 28 Bone Metabolism Unit, Internal Medicine Unit, Hospital General Universitari d’Elx, Carrer Almazara, 11, 03203 Elche, Alicante Spain; 29 Complejo Hospitalario de Pontevedra, Av Montecelo, 0, 36164, Casas Novas, Pontevedra, Spain; 30 Geriatric Unit, Hospital de Salamanca, Paseo de San Vicente, 139, 37007 Salamanca, Spain; 31 Geriatric Unit, Hospital Universitario de Getafe, Carr. De Madrid - Toledo, Km 12,500, 28905 Getafe, Madrid Spain; 32 Department of Surgery, Aragón Health Research Institute, University of Zaragoza, Zaragoza, Spain; 33 Musculoskeletal Research Unit, IMIM-Parc Salut Mar, CIBERFES, Universitat Autònoma de Barcelona, Doctor Aiguader 88, 08003 Barcelona, Spain

**Keywords:** Fragility hip fracture, Osteoporosis, Registries, Quality of life

## Abstract

**Purpose:**

The medical morbidity and mortality associated with neck of femur fractures is well-documented, whereas there is limited data for patient-reported outcomes. The aim of this study was to characterize the impact of neck of femur fractures on activities of daily living and patient-reported health-related quality of life.

**Methods:**

Design and participants: Multicentric prospective cohort study. Consecutive sample patients with fragility hip fracture over 50 years old admitted in 48 hospitals in Spain.

Outcomes: daily living activity function (Barthel Index) and health-related quality of life (EQ-5D) pre-fracture, admission to hospital and at 1- and 4-month follow-up post-fracture.

Statistics: Barthel and EQ-5D over time are described as mean (SD) and median (interquartile range).

**Results:**

A total of 997 patients were recruited at baseline with 4-month outcomes available for, and 856 patients (89.5%). Barthel Index fell from 78.77 (23.75) at baseline to 43.62 (19.86) on admission to hospital with the fracture. Scores partially recovered to 54.89 (25.40) and 64.09 (21.35) at 1- and 4-month post-fracture, respectively. EQ-5D fell from a median of 0.75 (0.47–0.91) to − 0.01 (− 0.03 to 0.51) on admission. Partial recovery was observed again to (0.51 (− 0.06 to 0.67)) and (0.60 (0.10 to 0.80)) at 1- and 4-month post-fracture, respectively.

**Conclusions:**

Hip fracture results in a large decline in the ability to perform activities of daily living and patient-reported health-related quality of life with only partial recovery amongst survivors 4-month post-fracture.

## Introduction

A total of 620,000 hip fractures were sustained in the European Union in 2010.Osteoporotic hip fractures are associated with significant morbidity, mortality and societal costs [1]. Fragility fractures of any site had an estimated economic burden of € 37 billion and accounted for almost 1.2 million quality-adjusted life years in that same year [2].

It is estimated that between 2020 and 2050, the number of hip fractures worldwide will increase to more than 2 billion cases [3]. This will represent a large socioeconomic burden [4, 5].

Despite the increasing socioeconomic burden, there is a scarcity of data on the impact of hip fracture to patients in terms of patient-reported outcomes and activities of daily living. Particularly, there is no previous reports to our knowledge of both in a same cohort in Spain. This is important for a comprehensive assessment of the burden of hip fractures in the Spanish population.

The aim of this study is to determine the association between proximal femur fragility fractures and patient-reported health-related quality of life and activities of daily living during the 4-month following fracture.

## Methods

### Study design and setting

Multi-centre prospective observational cohort study in 48 hospitals in Spain

### Eligibility criteria

Details on sampling strategies, data collection and follow-up have been reported elsewhere [6]. One thousand consecutive men or women aged ≥ 50 years old with a diagnosis of a fragility femur fracture were recruited. Consent was obtained from patient or principal carer and/or legal representative of the patient.

### Measurements

Data was collected during consultations on admission and at 1-month and 4-month follow-up appointments. Phone consultations were conducted for the 1 month and 4 months when face-to-face visits were not possible. Baseline (pre-fracture) measures were collected on admission to hospital-based on patient recollection of their previous activity and health status.

Outcome measures were Barthel Index (ability to perform activities of daily living) [7]) and EQ-5D-3L (global health-related quality of life [8]). Barthel scores range from 0 to 100, with lower scores indicating more disability. EQ-5D-3L measures five dimensions (mobility, self-care, usual activities, pain and anxiety/depression) and a global visual analogue scale (VAS). Utility indices were derived from EQ-5D-3L using Spanish national preference tables [9]. These range from 0 (death) or even negative values (worse than death) to 1 (full health).

### Statistical analyses

**Fig. 1:**
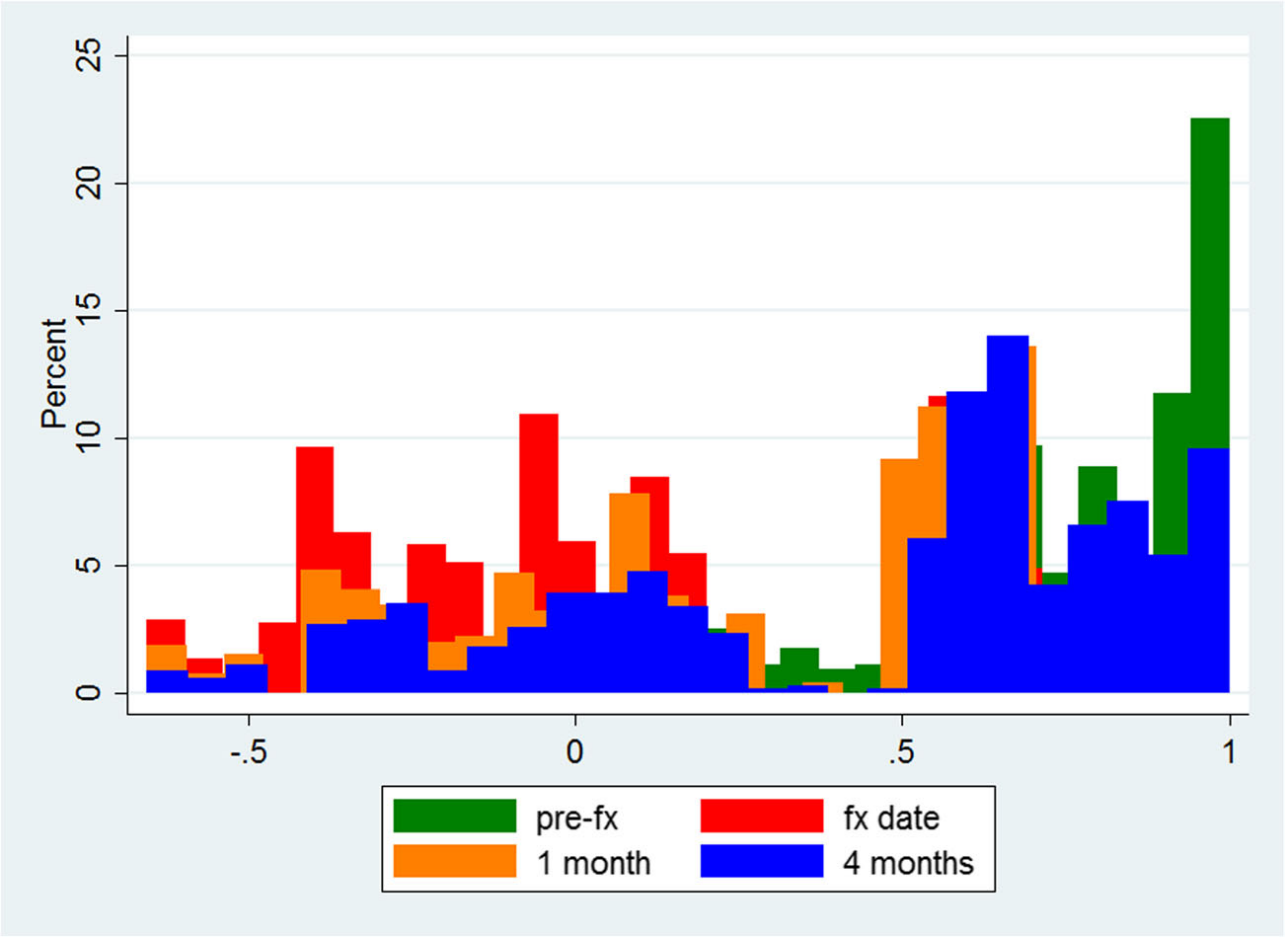
Histogram of EQ-5D-3L utility indices at baseline (pre-fx), at admission for a femur fracture (fx date), and at 1 and 4 months of follow-up

Barthel EQ-5D-3L is expressed as mean (standard deviation) and as median (interquartile range) (Table 1). Change in utility indices over time is plotted as a histogram (Fig. 1).

**Table 1:** Change in health-related quality of life at the time of a femur fracture, and in the following 1 and 4 months of follow-up

	Change in EQ-5D-3L utility at index admission	Change in EQ-5D-3L from index admission to 1 month	Change in EQ-5D-3L from 1 to 4 months of follow-up
Mean (sd)	− 0.57 (0.43)	0.23 (0.40)	0.37 (0.43)
Median (IQR)	− 0.56 (− 0.90 to − 0.28)	0.16 (0.0 to 0.50)	0.34 (0.09 to 0.70)

*Positive values are equivalent to improvement/recovery; negative values mean worsening

## Results

Baseline outcome data was available for 997 patients, of whom 856 (85.9%) completed 4 months of follow-up. Of the 141 without follow-up data, 99 (9.9%) have died, and 42 (4.2%) were lost to follow-up. Detailed patient characteristics have been previously reported [6]. Complete data on baseline and 1- and 4-month Barthel and EQ-5D-3L were collected for 824/856 (96.3%) and 746/856 (90.2%) participants respectively.

On average, overall health-related quality of life dropped by 57% at the time of fracture, to then recover by 23% in the first month and by a total 37% at 4-month post-admission.

## Discussion

Our study demonstrates a significant fall in both EQ-5D and the Barthel Index on sustaining a proximal femoral fracture, and this falls only partial recovers at 4-week follow-up. Barthel Index, assessing the ability to perform activities of daily living fell from 79 to 44% immediately after sustaining the fracture, with a recovery to 64% function 4-month post-fracture. The impact on patient quality of life is even more striking: participants started with 75% of full health which fell to 0% of full health ‘like dead’ immediately after the fracture. Partial recovery was again seen in the following 4 months, increasing to a 60% full health.

Our results are similar to those found in similar cohort from the UK [10], Norway [11] and Portugal [5]. In those, the authors reported a gradual recovery up to 1 year in terms of quality of life. These findings are also comparable to those obtained from a smaller Spanish cohort [12]. As in our study, improvement in quality of life seemed to be mirrored by functional recovery in these previous studies. Other studies [13, 14] including the UK (WHiTE) study [15] have also shown similar deterioration in functional status [16].

Our study has limitations. Our findings are only representative of the subjects who survived, as almost 10% of the study participants died within 4 months, and it is possible that their function and health status were worse at baseline and post-fracture. In addition, recall bias is possible in the determination of pre-fracture (baseline) health status, as this was recorded during the index hospital admission.

This analysis also has strengths. First, the prospective nature of data collection and the fact that the information was recorded by trained clinician/s and/or nurses give high validity to our findings. Secondly, we have low attrition, with < 5% loss to follow-up at 4-month post-fracture. Finally, our participants were recruited from a representative sample of hospitals around the country.

In conclusion, we report a sustained detriment of proximal femoral fractures on the ability of a patient to perform activities of daily living and their health-related quality of life. These data should be used to estimate the socioeconomic burden of osteoporosis-related fractures and to inform the planning of care for these patients.
